# New Cytotoxic Cyclic Peptide from the Marine Sponge-Associated *Nocardiopsis* sp. UR67

**DOI:** 10.3390/md16090290

**Published:** 2018-08-21

**Authors:** Alyaa Hatem Ibrahim, Eman Zekry Attia, Dina Hajjar, Mohamed A. Anany, Samar Yehia Desoukey, Mostafa Ahmed Fouad, Mohamed Salah Kamel, Harald Wajant, Tobias A. M. Gulder, Usama Ramadan Abdelmohsen

**Affiliations:** 1 Department of Pharmacognosy, Faculty of Pharmacy, Sohag University, 82524 Sohag, Egypt; dralyaahatem@gmail.com; 2 Department of Pharmacognosy, Faculty of Pharmacy, Minia University, 61519 Minia, Egypt; eman_zekry@mu.edu.eg (E.Z.A.); drsamaryehia@gmail.com (S.Y.D.); m_fouad2000@yahoo.com (M.A.F.); 3 Department of Biochemistry, Faculty of Science, Center for Science and Medical Research, University of Jeddah, 80203 Jeddah, Saudi Arabia; dhajjar@uj.edu.sa; 4 Division of Molecular Internal Medicine, Department of Internal Medicine II, University Hospital Würzburg, Röntenring 11, 97070 Würzburg, Germany; Mohamed_M@klinik.uni-wuerzburg.de (M.A.A.); harald.wajant@uni-wuerzburg.de (H.W.); 5 Division of Genetic Engineering and Biotechnology, Department of Microbial Biotechnology, National Research Centre, El Buhouth St., Dokki, 12622 Giza, Egypt; 6 Department of Pharmacognosy, Faculty of Pharmacy, Deraya University, Universities Zone, 61111 New Minia City, Egypt; mskamel@yahoo.com; 7 Biosystems Chemistry, Department of Chemistry and Center for Integrated Protein Science Munich (CIPSM), Technical University of Munich, Lichtenbergstraβe 4, 85748 Garching, Germany

**Keywords:** *Nocardiopsis*, cyclic hexapeptide, cytotoxicity, marine actinomycetes, sponges

## Abstract

A new cyclic hexapeptide, nocardiotide A (**1**), together with three known compounds—tryptophan (**2**), kynurenic acid (**3**), and 4-amino-3-methoxy benzoic acid (**4**)—were isolated and identified from the broth culture of *Nocardiopsis* sp. UR67 strain associated with the marine sponge *Callyspongia* sp. from the Red Sea. The structure elucidation of the isolated compounds were determined based on detailed spectroscopic data including ^1^D and ^2^D nuclear magnetic resonance (NMR) experimental analyses in combination with high resolution electrospray ionization mass spectrometry (HR-ESI-MS), while the absolute stereochemistry of all amino acids components of nocardiotide A (**1**) was deduced using Marfey’s method. Additionally, ten known metabolites were dereplicated using HR-ESI-MS analysis. Nocardiotide A (**1**) displayed significant cytotoxic effects towards the murine CT26 colon carcinoma, human HeLa cervix carcinoma, and human MM.1S multiple myeloma cell lines. The results obtained revealed sponge-associated *Nocardiopsis* as a substantial source of lead natural products with pronounced pharmacological activities.

## 1. Introduction

Actinomycetes are a diverse group of aerobic Gram-positive microorganisms withhigh guanine-cytosine DNA content [1]. They belong to the phylum Actinobacteria, which is one of the largest bacterial phyla, distributed in both terrestrial and marine ecosystems [2,3]. About 70% of all naturally derived drugs in clinical use originate from Actinobacteria as they contain biologically active secondary metabolites accounting for their clinical use, mainly as antibacterial, antifungal, antiviral, and cytotoxic drugs [4,5,6]. The genus *Nocardiopsis* was first described by Mayer in 1976 [7] as belonging to the family Nocardiopsaceae and as morphologically similar to members of the genera *Actinomadura* and *Nocardia* [7,8]. By reviewing the literature on the genus *Nocardiopsis* [9,10], it has been clearly demonstrated that it is a prolific producer of a wide variety of bioactive compounds, mainly cyclic peptides [11,12], polyketides [13,14], macrolides [15], alkaloids [16], diketopiperazines [17,18], α and γ-pyrones [19,20], naphthoquinones [21], phenazines [22], and phenoxazine derivatives [23], which contributes to a broad spectrum of biological activities, mainly as cytotoxic [21], anticancer [22], antitumor [24], antibacterial [11], antifungal [25], immunemodulatory [15],and protein kinase inhibitory [26].

Cancer still remains one of the most serious challenges to human health. Despite intense efforts to develop treatments, effective—particularly highly selective—agents are still not available for many cancer types. Therefore, it is necessary to continue the discovery of new classes of molecules with cytotoxic activity. One strategy to treat cancer is to find compounds with new scaffolds that have increased chances of possessing novel binding modes or even addressing novel targets. Consequently, this current investigation is a continuation of our efforts to seek new, effective cytotoxic agents from actinomycetes—associated with marine sponges, specifically, the *Nocardiopsis* sp. UR67 strain—and to evaluate their cytotoxic biological activities.

## 2. Results and Discussion

**Figure 1:**
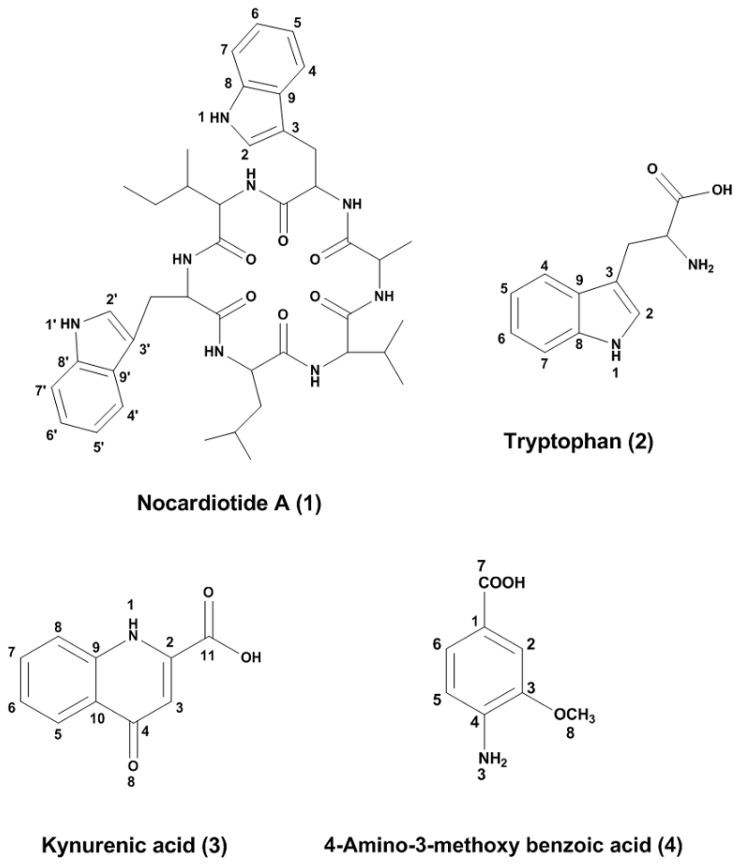
Structures of the isolated compounds.

*Nocardiopsis* sp. UR67 was cultivated from the sponge *Callyspongia* sp. (family Callyspongiidae) that was collected from the Red Sea (Ras Mohamed, Sinai, Egypt; (GPS: 27°47.655′ N; 34°12.904′ W) in August 2008. ISP2 liquid broth with calcium alginate beads [27] of *Nocardiopsis* sp. UR67 was extracted with ethyl acetate, and the obtained organic extract was fractionated on Sephadex LH20. This was followed by purification using semi-preparative reversed phase high performance liquid chromatography (HPLC) to yield a new cyclic hexapeptide nocardiotide A (**1**), along with three known compounds—tryptophan (**2**), kynurenic acid (**3**), which was isolated for the first time from microbial origins, and 4-amino-3-methoxy benzoic acid (**4**) (Figure 1).

### 2.1. Metabolomic Analysis

**Figure 2:**
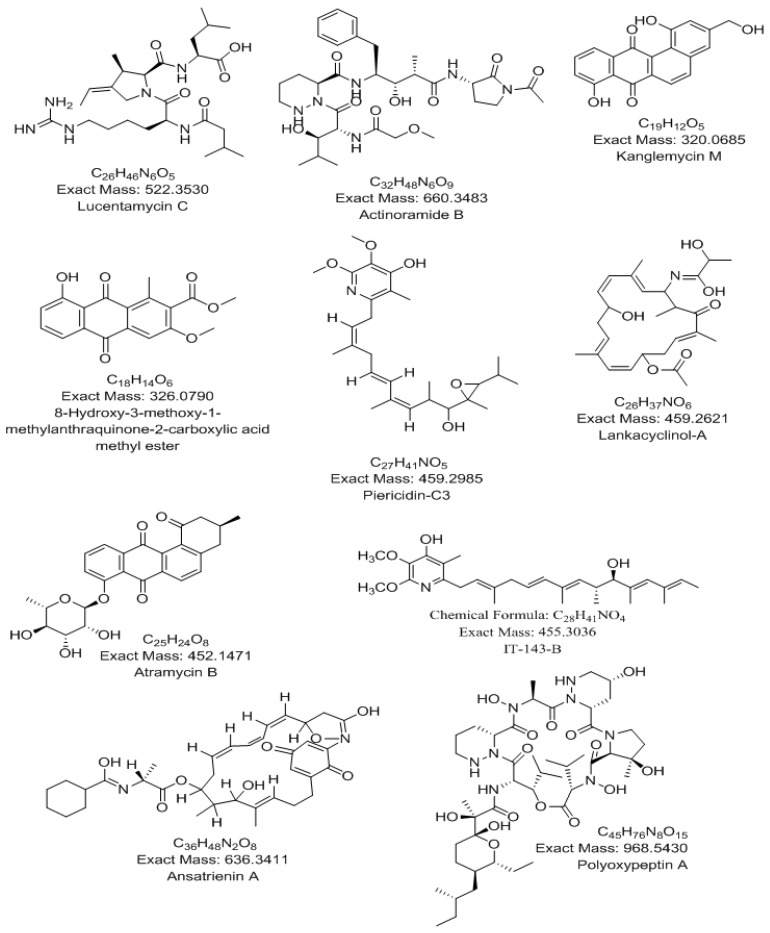
Dereplicated metabolites from metabolomic analysis of *Nocardiopsis* sp. UR67.

HPLC high resolution electrospray ionization mass spectrometry (HPLC-HR-ESIMS) analysis for dereplication purpose was used for identification of the metabolites from the ethyl acetate extract obtained from the culture broth of *Nocardiopsis* sp. UR67. The dereplication study of the metabolites (Figure 2, Table 1) against the Dictionary of Natural Products (DNP) database, AntiMarin, and METLIN databases led to the characterization of the following natural products: cytotoxic peptide lucentamycin C [28], immunosuppressant kanglemycin M [29], 8-hydroxy-3-methoxy-1-methyl-anthraquinone-2-carboxylic acid [30], antimicrobial, antitumor and insecticidal piericidin-C3 [31], sotetracenone-type antitumor atramycin B [32], piericidin group antibiotic IT-143-B [33], antibiotic lankacyclinol-A [34], antifungal polyketide ansatrienin A [35], actinoramide B [36] and, finally, a potent apoptosis inducer polyoxypeptin A [37].

### 2.2. Structure Elucidation

**Figure 3:**
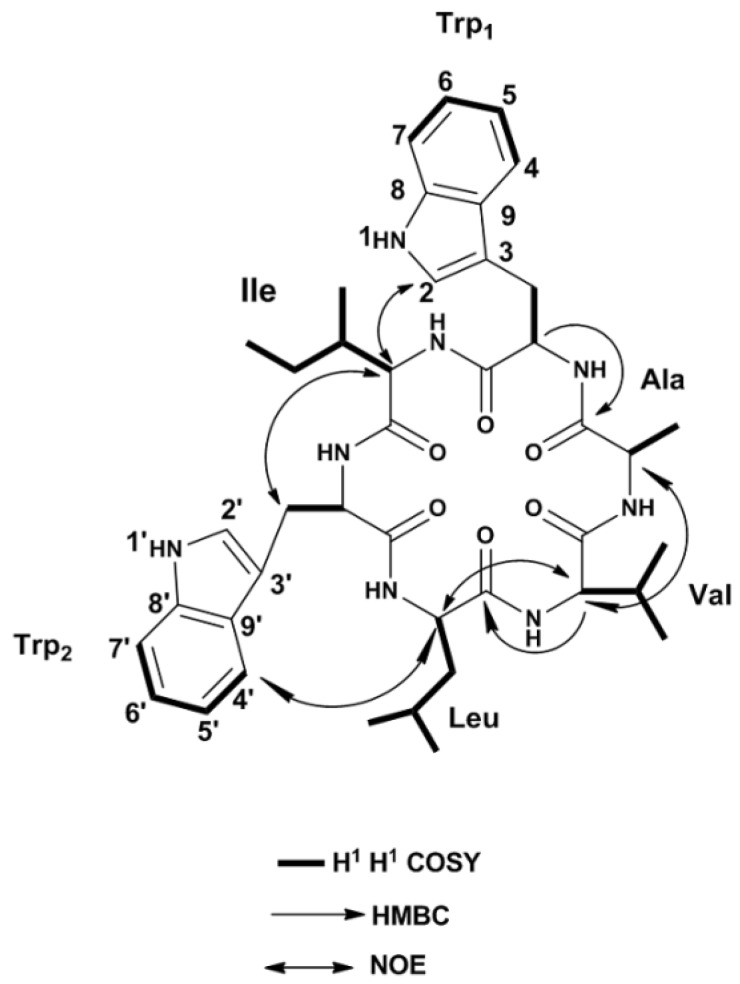
Significant COSY, heteronuclear multiple bond correlation (HMBC), and NOE correlations of nocardiotide A (**1**).

Nocardiotide A (**1**) was obtained as a pale yellow powder with a molecular formula of C_42_H_56_N_8_O_6_ determined by HR-ESI-MS analysis (*m*/*z* 791.931 [M + Na]^+^, calcd. for C_42_H_56_N_8_O_6_Na), indicating 19 degrees of unsaturation. The peptidic nature of nocardiotide A (**1**) was recognized from the ^1^H and ^13^C nuclear magnetic resonance (NMR) spectral data (Table 2). The ^1^H-NMR spectrum (Figures S1–S3) revealed the presence of six α-amino acid hydrogen resonances (δ_H_ 3.35–4.37). Additionally, the ^13^C NMR spectrum (Figure S4) contained six amide carbonyl signals resonating between δ_C_ 171 and 179 ppm and six α-amino acid carbon signals between δ_C_ 41 and 60 ppm, thus corroborating the presence of six amino acid moieties [38,39]. The ^1^H-NMR and COSY spectra (Figures S1 and S7) showed two distinct aromatic spin systems (δ_H_ 6.85–7.55), and the ^13^C-NMR and ^13^C-DEPT-135 spectrum (Figures S4 and S5) displayed ten methines and six quaternary carbons consistent with two tryptophan moieties. One tryptophan (Trp_1_) was assigned at δ_H_ 4.37 (dd, *J* = 3.8, 9.9 Hz, H1-α), 3.30 (dd, *J* = 14.7, 3.8 Hz, H1-β), 3.05 (dd, *J* = 14.7, 9.98 Hz, H1-β′), 7.03 (s, H1-2), 7.55 (dt, *J* = 7.84, 0.9 Hz, H1-4), 6.88 (td, overlapped, H1-5), 6.94 (td, overlapped, H1-6), 7.18 (dt, *J* = 8.11, 0.9 Hz, H1-7), and their corresponding carbons signals were assigned at δ_C_ 179.06 (CO1), 56.90 (C1-α), 29.19 (C1-β), 124.65 (C1-2), 112.29 (C1-3), 119.55 (C1-4), 119.44 (C1-5), 122.05 (C1-6), 112.04 (C1-7), 137.98 (C1-8), 128.92 (C1-9). The other tryptophan residue (Trp_2_) was assigned at δ_H_ 3.35 (t, *J* = 8.04 Hz, H2-α), 2.82 (td, *J* = 8.64, 0.9 Hz, H2-β), 6.96 (s, H2-2′), 7.45 (dt, *J* = 7.92, 0.9 Hz, H2-4′), 6.90 (td, overlapped, H2-5′), 6.98 (td, overlapped, H2-6′), 7.22 (dt, *J* = 8.17, 0.9 Hz, H2-7′), while their corresponding carbons signals were detected at δ_C_ 173.24 (CO2), 41.56 (C2-α), 26.21 (C2-β), 123.33 (C2-2′), 113.27 (C2-3′), 119.22 (C2-4′), 119.44 (C2-5′), 122.28 (C2-6′), 112.20 (C2-7′), 138.17 (C2-8′), 128.80 (C2-9′). These assignments were further confirmed by heteronuclear single quantum coherence (HSQC) and heteronuclear multiple bond correlation (HMBC) correlations (Figure 3) [40]. In addition to these two tryptophan moieties, the ^13^C-NMR, ^13^C-DEPT-135, and HSQC spectra (Figures S4–S6), displayed seven methyl, two methylene, and seven methine carbons. A spin system characteristic for isoleucine was observed at δ_H_ 4.20 (d, *J* = 3.9 Hz, H-α), 2.03 (m, H-β), 0.84 (d, *J* = 7.0 Hz, H-γ), 1.28 (m, H-γ′), and 0.85 (d, *J* = 7.4 Hz, H-δ), while their corresponding carbon signals were observed at δ_C_ 58.94 (C-α), 36.93 (C-β), 12.14 (C-γ), 27.49 (C-γ′), and 14.65 (C-δ), respectively. The previous assignments were corroborated using HSQC experiment (Figure S6). In addition, HMBC correlation (Figure S10) from α-proton of isoleucine (δ_H_ 4.20, d, *J* = 3.9 Hz) to its own amide-type carbonyl at δ_C_ 173.42 was detected. Furthermore, an alanine spin system was displayed at δ_H_ 4.31 (q, *J* = 7.2 Hz, H-α), and 1.17 (d, *J* = 7.2 Hz, H-β), and their corresponding carbons were observed at δ_C_ 49.77 (C-α) and 18.05 (C-β), respectively. The α and β protons of alaninemoiety (δ_H_ 4.31 and 1.17, respectively) showed a strong HMBC correlations with their amide-type carbonyl at δ_C_ 173.93. Additionally, a spin system for a leucine residue was observed at δ_H_ 3.81 (t, *J* = 7.3 Hz, H-α), 1.59 (m, H-β), 1.62 (m, H-γ), 0.92 (d, *J* = 6.2 Hz, H-δ), and 0.93 (d, *J* = 6.2 Hz, H-δ′), and their corresponding carbons were assigned at δ_C_ 52.97 (C-α), 41.95 (C-β), 25.60 (C-γ), 22.56 (C-δ), and 22.85 (C-δ′), respectively, using HSQC experiment correlation. Moreover, the α and β protons of leucine residue exhibited HMBC correlations with their amide-type carbonyl at δ_C_ 171.56. Finally, a valine moiety was detected from the spin system at δ_H_ 4.22 (d, *J* = 7.7 Hz, H-α), 1.97 (m, H-β), 0.88 (d, *J* = 3.1 Hz, H-γ), and 0.89 (d, *J* = 3.1 Hz, H-γ′), and their corresponding carbons were displayed at δ_C_ 60.76 (C-α), 31.75 (C-β), 19.79 (C-γ), and 18.95 (C-γ′), respectively. The amide-type carbonyl at δ_C_ 175.33 was attributed to the valine residue, which could be confirmed from the strong HMBC correlations observed amongst α and β protons of valine moiety [40]. Detailed analysis of the ^1^D (^1^H, ^13^C and DEPT-135) and ^2^D (HSQC, HMBC and NOE) NMR spectroscopic data (Table 2) revealed that nocardiotide A (**1**) was a hexapeptide containing Ile, Leu, Val, Ala, and two Trp residues. The amino acid sequence was elucidated to be Ile-Trp_1_-Ala-Val-Leu-Trp_2_ on the basis of the following HMBC correlations (Figures S10 and S11): α-Trp_1_ (δ_H_ 4.37)/Ala-CO (δ_C_ 173.9), α-Val (δ_H_ 4.22)/Leu-CO (δ_C_ 171.5) and the following NOE correlations (Figures S8 and S9): Trp_1_H-2(δ_H_ 7.02)/α-Ile (δ_H_ 4.20), α-Ala (δ_H_ 4.31)/α-Val (δ_H_ 4.22), α-Val (δ_H_ 4.22)/α-Leu (δ_H_ 3.81), α-Ile (δ_H_ 4.20)/β,β′ Trp_2_ (δ_H_ 2.82) and Trp_2_ H-4′ (δ_H_ 7.45)/α-Leu (δ_H_ 3.81) (Table 2, Figure 3) [38,41].

These six amino acids accounted for18 degrees of unsaturation, indicating that nocardiotide A (**1**) was a monocyclic hexapeptide. The absolute configurations of the amino acid units in nocardiotide A (**1**) were determined by acid hydrolysis, followed by chiral derivatization with Marfey’s reagent (1-fluoro-2,4-dinitrophenyl-5-lalanine amide, FDAA). HPLC analysis of the Marfey’s derivatives in comparison to their respective d-and l-authentic reference amino acids revealed the absolute configuration of all amino acids of the new cyclic hexapeptideto beL.

Additionally, three known compounds—tryptophan (**2**), kynurenic acid (**3**), and 4-amino-3-methoxy benzoic acid (**4**) (Figure 1)—were also separated and could be identified by comparing their ^1^D and ^2^D NMR spectral analysis (Figures S12–S26) with the published data [42,43]. It is worth mentioning that kynurenic acid (**3**) was isolated for the first time from microbial origins.

### 2.3. Biological Activities of the Isolated Compounds

**Figure 4:**
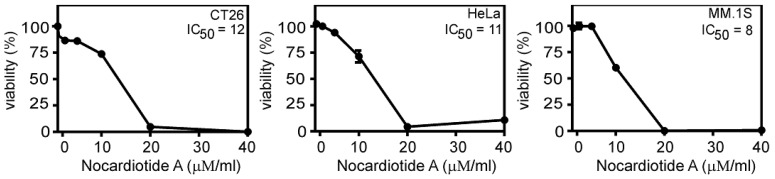
Nocardiotide A induces cell death in CT26, HeLa, and MM.1S cell lines.

The four aforementioned isolated compounds were examined for their cytotoxicity potential towards the murine CT26 colon carcinoma, the human HeLa cervix carcinoma, and the human MM.1S multiple myeloma cell lines. Nocartiodite A (**1**) displayedprominent cytotoxic features with IC_50_ values of 8, 11, and 12 μM/mL against the human MM. 1S multiple myeloma, human HeLa cervix carcinoma, and murine CT26 colon carcinoma, respectively (Figure 4). Tryptophan (**2**), kynurenic acid (**3**), and4-amino-3-methoxy benzoic acid (**4**) did not demonstrate any considerable cell death properties at the examined concentration.

## 3. Materials and Methods

### 3.1. General Experimental Procedures

Melting points were measured using Stuart Scientific (SMPI) melting point apparatus and were uncorrected. An ultraviolet lamp (CAMAG, Wilmington, NC, USA) was used for visualization of spots on thin layer chromatograms at 254 and/or 365 nm. ^1^H (600 MHz) and ^13^C (150 MHz) NMR spectra were recorded on BrukerAvance III HD 600 instruments (Bruker Biospin, Rheinstetten, Germany) in CD_3_OD. The samples were degassed by an ultrasonic water bath (Branson 3800 Ultrasonic Cleaner, Branson, Gayton, UK) for 20 min before measurements. Solvent signals of CD_3_OD (δ_H_ 3.3 ppm and δ_C_ 49.0 ppm) were considered as the internal reference signals for calibration. Chemical shift values (δ) were recorded in ppm units and coupling constants (*J*) in Hz. Heteronuclear correlations were measured using HSQC (optimized for 1 *J*_HC_ = 145 Hz) and HMBC (optimized for n *J*_HC_ = 8.3 Hz or n *J*_HC_ = 4.0 Hz) pulse sequences. Positive and negative HR-ESI-MS spectra were obtained using a Synapt G2 HDMS QTOF-mass spectrometer (Waters, Eschborn, Germany). HPLC separations and purifications were performed on the Knauer system (Knauer, Berlin, Germany). This included Smartline S-1000 quaternary pumps coupled with a Smartline S-2600 UV–VIS multiwavelength detector (Knauer, Berlin, Germany), a Knauer dynamic mixing chamber, and using a C18 column (5 µm, 10 mm × 250 mm, Knauer, Berlin, Germany) at ambient temperature with a guard column filled with the same stationary phase. On the other hand, the analytical detection was carried out using an analytical Gemini-NX RP-18 column (5 µm, 4.60 mm × 100 mm; Phenomenex, Aschaffenburg, Germany).

### 3.2. Sponge Collection

*Callyspongia* sp. (family Callyspongiidae) was collected at a depth of 10 m in the Red Sea (Ras Mohamed, Sinai, Egypt; (GPS: 27°47.655′ N; 34°12.904′ W) in August 2008. The collected sponge was transferred to plastic bags containing seawater and transported to the laboratory. The sponge was identified by R.W.M. van Soest (University of Amsterdam, Amsterdam, The Netherlands).

### 3.3. Isolation, Fermentation, and Extract Preparation of Nocardiopsis sp. UR67

Sponge specimens were rinsed in sterile seawater, cut into pieces of ca. 1 cm^3^, and then thoroughly homogenized in a sterile mortar with 10 volumes of sterile seawater. The supernatant was diluted in a tenfold series (10^−1^, 10^−2^, 10^−3^) and subsequently plated out on agar plates. Four different media—M1, ISP medium 2, Oligotrophic medium (OLIGO), and Marine Agar (MA)—were used for the isolation of actinobacteria. All media were supplemented with 0.2 µm pore size filtered cycloheximide (100 µg/mL), nystatin (25 µg/mL), and nalidixic acid (25 µg/mL) to facilitate the isolation of slow-growing actinobacteria; cycloheximide and nystatininhibit fungal growth, while nalidixic acid inhibits many fast-growing Gram-negative bacteria [44]. All media contained DifcoBacto agar (18 g/L) and were prepared in 1 L artificial sea water (NaCl 234.7 g, MgCl_2_·6 H_2_O 106.4 g, Na_2_SO_4_ 39.2 g, CaCl_2_ 11.0 g, NaHCO_3_ 1.92 g, KCl 6.64 g, KBr 0.96 g, H_3_BO_3_ 0.26 g, SrCl_2_ 0.24 g, NaF 0.03 g, and ddH_2_O to 10.0 L). The inoculated plates were incubated at 30 °C for 6–8 weeks. Distinct colony morphotypes were picked and restreaked until visually free of contaminants. The isolates were maintained on plates for short-term storage and long-term strain collections. *Nocardiopsis* sp. UR67 was fermented in 10 Erlenmeyer flasks (2 L), each containing 1 L of ISP 2 (International Streptomyces Project) medium in artificial sea water and incubated at 30 °C for 10 days with shaking at 150 rpm. After fermentation and filtration, the supernatant was extracted with ethyl acetate (3 × 500 mL) to give the organic extract for subsequent compound isolation.

### 3.4. LC-HR/MS Analysis

Ethyl acetate extract of 1 mg/mL in MeOH was analyzed on an Accela HPLC (Thermo Scientific, Karlsruhe, Germany) coupled to a UV detector at 280 and 360 nm and an Exactive-Orbitrap high resolution mass spectrometer (Thermo Fisher Scientific, Karlsruhe, Germany). The HPLC column was an ACE (ACE, Mainz, Germany) C18, 75 mm × 3.0 mm, 5 μm column. The mobile phase consisted of purified water (A) and acetonitrile (B) with 0.1% formic acid in each solvent. The gradient program started with 10% B linearly increased to 100% B at a flow rate of 300 µL/min for 30 min and remained isocratic for 5 min before linearly decreasing back to 10% B in 1 min. The column was then re-equilibrated with 10% B for 9 min before the next injection. The total analysis time for each sample was 45 min. The injection volume was 10 µL, and the tray temperature was maintained at 12 °C. High resolution mass spectrometry was carried out in both positive and negative ESI ionization modes with a spray voltage at 4.5 kV and capillary temperature at 320 °C. The mass range was set from *m*/*z* 150–1500. Both negative and positive ionization switch modes were used to include the highest number of metabolites from the investigated bacterial fractions subjected to LC–HR-ESIMS analysis. The dereplication was achieved for each *m*/*z* ion peak with metabolites recorded in the customized databases based on established parameters (*m*/*z* threshold of ±3 ppm and retention time) [45], which provided a high level of confidence in metabolites identity; consequently, the number of the remaining unknown metabolites in each bacterial fraction was refined.

### 3.5. Metabolites Isolation

Theethyl acetateextract (5 g) was fractionated on a silica gel (250 g, 15–25 μm, 120 cm × 2.5 cm, Merck, Darmstadt, Germany) column and eluted with a DCM/MeOH gradient from (90:10%) to 100% methanol. The effluents were collected in fractions (50 mL each). Similar fractions monitored by TLC were combined and concentrated to yield six raw fractions. Fraction 2 (850 mg) was further fractionated bySephadex LH-20 column chromatography (50 g, 32–64 µm, 120 cm × 2 cm, Merck, Darmstadt, Germany) eluted with MeOH to yield five subfractions. Subfractions II and IV (compounds-rich subfractions) were further purified by semi-preparative HPLC using H_2_O/acetonitrile(90:10%) initially for 10 min, then by a linear gradient to 100% acetonitrilewithin 60 min, which was then followed by an isocratic elution at 100% acetonitrilefor a further 10 min with a flow rate of 2.0 mL/min using a C18 column (5 µm, 10 mm × 250 mm, Knauer, Berlin, Germany) to yield compound **1** (4 mg; *R*_t_ = 14.3 min) and compound **2** (3 mg; *R*_t_ = 16.5 min) from subfraction II as well as compound **3** (1 mg; *R*_t_ = 17.1 min) and compound **4** (2 mg; *R*_t_ = 17.9 min) from subfraction IV.

### 3.6. Marfey’s Analysis

The absolute configurations of the amino acids in compound **1** were elucidated by Marfey’s derivatization and compared to the corresponding standard amino acids each with D and L configurations (Sigma, Darmstadt, Germany) by HPLC. Compound **1** (1 mg) was initially hydrolyzed with 6 M HCl (2 mL) in a water bath at 100 °C for 24 h. The hydrolysate was cooled to room temperature, dried using a vacuum evaporator and dissolved in 100 μL of water. The Marfey’s derivatization was carried out by adding 100 μL of 1% Marfey’s reagent (1-fluoro-2,4-dinitrophenyl-5-l-alanine amid) dissolved in acetone and 20 μL of 1 M NaHCO_3_ (H_2_O) to 50 μL of the hydrolysate of compound **1** as well as 50 mM standard amino acid, respectively, and incubated at 40 °C for 1 h with frequent shaking. The reaction was stopped by adding 10 μL of 2 M HCl after cooling. The Marfey’s derivatization products were finally dried and prepared in MeOH for further HPLC analysis. The HPLC chromatography was carried out on Gemini-NX RP-C18 column by eluting with H_2_O/acetonitrile (95:5%) for the first 5 min, linearly gradient to 100% acetonitrile for 30 min, and staying at 100% actonitrile for a further 10 min with a flow rate at 1 mL/min and UV detection at 340 nm. The configuration was eventually determined with the observation of the same retention times compared to the standard enantiomeric amino acids [46,47,48]. Retention times (min) of authentic amino acids were as follows: l-Val (25.4), d-Val (27.3), l-Leu (21.9), d-Leu (22.2), l-Ala (18.7), d-Ala (20.3), l-Trp (27.3), d-Trp (29.6). A better resolution of the l-Ile, d-Ile derivatives was achieved using a linear gradient of acetonitrile in 0.1% (*v*/*v*) aqueous TFA (30–45% acetonitrile over 50 min): l-Ile (30.7), d-Ile (38.5).

### 3.7. Cytotoxic Activity

The cytotoxicity of the isolated compounds was evaluated in cell lines using the 3-(4,5-dimethylthiazol-2-yl)-2,5-diphenyltetrazolium bromide (MTT) assay. CT26, HeLa, and MM.1S cells were maintained in RPMI medium (Merck, Darmstadt, Germany), supplemented with 10% fetal bovine serum (FBS), and grown at 37 °C and 5% CO_2_. HeLa cells (2 × 10^4^ per well) were plated in 96-well tissue culture plates in 100 μ Lcell culture medium. The following day, cells were stimulated overnight in triplicates with the reagents of interest. Cell viability was assessed by crystal violet staining. In case of the CT26 and MM.1S cell lines, cells were seeded in 96-well plates (7× 10^4^cells per well) and were challenged the same day overnight with the reagents of interest; the cytotoxic effect was evaluated using the MTT assay [49]. To normalize cell viability values, each plate included a triplicate of cells treated with the compound carrier DMSO to define 100% viable cells as well as a triplicate of cells incubated with a cytotoxic mixture (200 ng/ML Tumor Necrosis Factor TNF, 200 ng/mL CD95L (Fas ligand), 200 ng/mL TRAIL (TNF-related apoptosis-inducing ligand), 25 µg/mL CHX (Cycloheximide), 1% (*w*/*v*) sodium azide) to define maximal cell death and thus 0% viability. All other viability values were normalized according to the averages of these triplicates and analyzed by the Graph Pad Prism 5 software (La Jolla, CA, USA).

### 3.8. Compounds Characterization

#### 3.8.1. (Nocardiotide A) (**1**)

Pale yellow solid (4 mg; *R*_t_ = 14.3 min) UV (EtOH) λ max 232, 305 nm; its ^1^H NMR (CD3OD, 600 MHz) and ^13^C NMR (CD3OD, 150 MHz) data aredetailed anddisplayed in Table 2.

#### 3.8.2. (Tryptophan) (**2**)

Pale yellow crystalline solid (3 mg; *R*_t_ = 16.5 min) (m.p. 283–285 °C); ^1^H NMR (CD3OD, 600 MHz): δ 7.20 (1H, s, H-2), 7.61 (1H, dt, *J* = 8.0, 1.0 Hz, H-4), 7.06 (1H, td, *J* = 7.6, 1.0 Hz, H-5), 7.14 (1H, td, *J* = 7.6, 1.0 Hz, H-6), 7.38 (1H, dt, *J* = 8.0, 0.96 Hz, H-7), 3.50 (1H, dd, *J* = 4.47, 15.3 Hz, H-10a), 3.33 (obscured by solvent, H-10b), 4.23 (1H, dd, *J* = 4.8, 8.1 Hz, H11); ^13^C NMR (CD3OD, 150 MHz): δ 125.4 (CH, C-2), 108.0 (C, C-3), 117.0 (CH, C-4), 120.3 (CH, C-5), 123.0 (CH, C-6), 112.6 (CH, C-7), 138.4 (C, C-8), 128.4 (C, C-9), 27.7 (CH2, C-10), 54.7 (CH, C-11), 171.9 (C, C-12). The physical and spectral data were in accordance with those reported in the literature [42].

#### 3.8.3. (Kynurenicacid) (**3**)

White amorphous powder; (1 mg; *R*_t_ = 17.1 min) ^1^H NMR (CD3OD, 600 MHz): δ 7.01 (1H, s, H-3), 8.26 (1H, ddd, *J* = 8.5, 1.4, 0.6 Hz, H-5), 7.47 (1H, td, *J* = 7.7, 1.4 Hz, H-6), 7.78 (1H, td, *J* = 7.7, 1.0 Hz, H-7), 7.88 (1H, ddd, *J* = 8.5, 1.0, 0.6 Hz, H-8); ^13^C NMR (CD3OD, 150 MHz): δ 143.3 (C, C-2), 110.3 (CH, C-3), 181.1 (C, C-4), 126.0 (CH, C-5), 126.0 (CH, C-6), 134.3 (CH, C-7), 120.4 (CH, C-8), 141.3 (C, C-9), 126.5 (C, C-10), 164.8 (C, C-11). The physical and spectral data were in accordance with those reported in the literature [43]. This is the first isolation of this compound from microbial origins.

#### 3.8.4. (4-Amino3-methoxy benzoic acid) (**4**)

Pale yellow crystalline powder (2 mg; *R*_t_ = 17.9 min) (m.p. 185–187 °C); ^1^H NMR (CD3OD, 600 MHz): δ 7.61 (1H, d, *J* = 1.2 Hz, H-2), 6.79 (1H, d, *J* = 9.8 Hz, H-5), 7.53 (1H, dd, *J* = 9.8, 1.2 Hz, H-6); ^13^C NMR (CD3OD, 150 MHz): δ 127.4 (C, C-1), 112.5 (CH, C-2), 146.8 (C, C-3), 149.3 (C, C-4), 113.9 (CH, C-5), 123.1 (CH, C-6). The physical and spectral data were in accordance with those reported in the literature.

## 4. Conclusions

In continuation of our interest to isolate and identify new antiproliferative agents from natural sources, the chemical characterization of *Nocardiopsis* sp.UR67—an actinomycete associated with the sponge (*Callyspongia* sp.) previously collected from the Red Sea—was conducted alongside with evaluation of the cytotoxic properties of the attained compounds versus the murine CT26 colon carcinoma, the human HeLa cervix carcinoma, and the human MM.1S multiple myeloma cell lines. Ten known metabolites were identified by dereplication using LC-HR-ESI-MS techniques. Additionally, four compounds were isolated and characterized for the first time from the broth culture of *Nocardiopsis* sp. UR67. Most importantly, one new cyclic hexapeptide—nocardiotide A—was identified, along with tryptophan, kynurenic acid, and 4-amino-3-methoxy benzoic acid. Among them, only nocardiotide A demonstrated significant cytotoxic property.

## Figures and Tables

**Table 1:** The dereplication results of the ethyl acetate fraction.

Polarity	*m*/*z*	Rt (min.)	Formula	Name	Source
[M + H]^+^	327.0866	2.91	C_18_H_14_O_6_	8-Hydroxy-3-methoxy-1-methylanthraquinone-2-carboxylic acid	*Streptomyces* sp.
[M − H]^−^	967.5399	3.53	C_45_H_76_N_8_O_15_	Polyoxypeptin A	*Streptomyces* sp. MK498-98 F14
[M − H]^−^	635.3316	3.81	C_36_H_48_N_2_O_8_	Ansatrienin A	*Streptomyces collinus*
[M + H]^+^	321.0760	4.6	C_19_H_12_O_5_	Kanglemycin M	*Nocardiamediterranei var. kanglensis* 1747-64
[M + H]^+^	661.3568	6.41	C_32_H_48_N_6_O_9_	Actinoramide B	*Streptomyces ballenaensis* and *Streptomyces bangulaensis*
[M + H]^+^	460.2697	6.95	C_26_H_37_NO_6_	Lankacyclinol-A	*Streptomyces rochei var. volubilis*
[M − H]^−^	451.1392	7.24	C_25_H2_4_O_8_	Atramycin B	*Streptomyces atratus*
[M − H]^−^	458.2906	7.56	C_27_H_41_NO_5_	Piericidin-C3	*Streptomyces pactum*
[M + H]^+^	456.3108	7.98	C_28_H_41_NO_4_	IT-143-B	*Streptomyces* species
[M + H]^+^	523.3601	11.23	C_26_H_46_N_6_O_5_	Lucentamycin C	*Nocardiopsis lucentensis*

**Table 2:** NMR-spectroscopic data of nocardiotide A (**1**) in MeOD-d_4_ (^1^H: 600 MHz; ^13^C: 150 MHz, *δ* in ppm, *J* in Hz).

Aminoacids	δ_C_	δ_H_, mult (J in Hz)	COSY	HMBC	NOESY
Ile					
CO	173.42				
α	58.94	4.20, d (4.6)	β	CO, β, γ′, δ	β-Trp_2_
β	36.93	2.03, m	α, γ, γ′	γ′, δ	
γ	12.14	0.84, d (7.0)	γ′, β		
γ′	27.49	1.28, m	β, γ, δ	α, β, γ, δ	
δ	14.65	0.85, d (7.4)	γ′	α, β, γ′	
Trp_1_					
CO	179.06				
α	56.90	4.37, dd (3.8, 9.9)	β, β′	CO, β/β′, C-3, Ala-CO	
β	29.19	3.05, dd (14.7, 9.9)	α	CO, α, C-2, C-3, C-8, C-9	
β′	29.19	3.30, dd (14.7, 3.8)	α		
2	124.65	7.03 (s)		β/β′, C-3, C-8, C-9	α-Ileu
3	112.29				
4	119.55	7.55, dt (7.84, 0.9)	H-5, H-6, H-7	C-3, C-6, C-8, C-9	
5	119.44	6.88 m	H-4, H-6, H-7	C-7, C-9	
6	122.05	6.94 (m)	H-4, H-5, H-7	C-4, C-9	
7	112.04	7.18, dt (8.11, 0.9)	H-4, H-5, H-6	C-5, C-9	
8	137.98				
9	128.92				
Ala					
CO	173.93				
α	49.77	4.31, q, (7.2)	β	CO, β	α-Val
β	18.05	1.17, d, (7.2)	α	CO, α	
Val					
CO	175.33				
α	60.76	4.22, d (7.7)	β	CO, Leu-CO, β, γ′	α-Leu
β	31.75	1.97, m	α, γ, γ′	CO, α, γ	
γ	19.79	0.88, d (3.1)	β	α, β, γ′	
γ′	18.95	0.9, d (3.1)	β	α, β, γ	
Leu					
CO	171.56				
α	52.97	3.81, t (7.3)	β	CO, β, γ	
β	41.95	1.59, m	α, γ	CO, α, γ, δ, δ′	
γ	25.60	1.62, m	β, δ, δ′	α, β, δ, δ′	
δ	22.56	0.92, d (6.2)	γ	β, γ, δ′	
δ′	22.85	0.93, d (6.2)	γ	β, γ, δ	
Trp_2_					
CO	173.24				
α	41.56	3.35, t (8.04)	β/β’	CO, β/β′, C-3′	
β/β′	26.21	2.82, td (8.64, 0.9)	α	α, C-2′, C-3′, C-9′	α-Ile
2′	123.33	6.96, s		α, β/β’	
3′	113.27				
4′	119.22	7.45, dt (7.92, 0.9)	H-5′, H-6′, H-7′	C-3′, C-6′, C-8′, C-9′	
5′	119.44	6.90, m	H-4′, H-6′, H-7′	C-7′, C-9′	
6′	122.28	6.98, m	H-4′, H-5′, H-7′	C-4′, C-9′	
7′	112.20	7.22, dt (8.17, 0.9)	H-4′, H-’5, H-6′	C-5′, C-9′	
8′	138.7				
9′	128.80				

## Supplementary Materials

The following are available online at http://www.mdpi.com/1660-3397/16/9/290/s1, Figures S1–S11: ^1^H-NMR, ^13^C-NMR, COSY, HSQC, NOESY and HMBC spectra of **1**, Figures S12–S17: ^1^H-NMR, ^13^C-NMR, COSY, HSQC and HMBC spectra of **2**, Figures S18–S21: ^1^H-NMR, ^13^C-NMR, COSY and HMBC spectra of **3**, Figures S22–S26: ^1^H-NMR, ^13^C-NMR, COSY, HSQC and HMBC spectra of **4**.
