# Supplementary material for: New Cytotoxic Cyclic Peptide from the Marine Sponge-Associated Nocardiopsis sp. UR67
Source: Mar Drugs. 2018 Aug 21;16(9):290. doi: 10.3390/md16090290 (PMC6174345; doi:10.3390/md16090290)
Supplement: Supplementary file 1 [file marinedrugs-16-00290-s001.zip › marinedrugs-343374-supp.docx]

(Article)

New Cytotoxic Cyclic peptide from the Marine Sponge-Associated Nocardiopsis sp. UR67

Alyaa Hatem Ibrahim^1^, Eman Zekry Attia^2^, Dina Hajjar^3^, Mohamed A. Anany^4,7^, Samar Yehia Desoukey ^2^, Mostafa Ahmed Fouad^2^, Mohamed Salah Kamel ^5^, Harald Wajant^4^, Tobias A. M. Gulder^6^*, Usama Ramadan Abdelmohsen^2^*

^1^ Department of Pharmacognosy, Faculty of Pharmacy, Sohag University, 82524 Sohag, Egypt; [dralyaahatem@gmail.com](mailto:dralyaahatem@gmail.com)

^2^ Department of Pharmacognosy, Faculty of Pharmacy, Minia University, 61519 Minia, Egypt; eman_zekry@mu.edu.eg(E.Z.A), [drsamaryehia@gmail.com](mailto:drsamaryehia@gmail.com) (S.Y.D), [m_fouad2000@yahoo.com](mailto:m_fouad2000@yahoo.com) (M.A.F) and [usama.ramadan@mu.edu.eg](mailto:usama.ramadan@mu.edu.eg) (U.R.A)

^3^ Department of Biochemistry, Faculty of Science, Center for Science and medical research, University of Jeddah, 80203 Jeddah, Saudi Arabia; [dhajjar@ju.edu.sa](mailto:dhajjar@ju.edu.sa)

^4^ Division of Molecular Internal Medicine, Department of Internal Medicine II, University Hospital Würzburg, Röntenring 11, 97070 Würzburg, Germany;harald.wajant@mail.uni-wuerzburg.de

^5^ Department of Pharmacognosy, Faculty of Pharmacy, Deraya University, Universities Zone, 61111 New

    Minia City, Minia, Egypt;[mskamel@yahoo.com](mailto:mskamel@yahoo.com) (M.S.K)

^6^ Department of Chemistry and Center for Integrated Protein Science Munich (CIPSM), Department of

   Chemistry, Biosystems Chemistry, Technical University of Munich, Lichtenbergstraβe 4, 85748 Garching,

   Germany; [tobias.gulder@ch.tum.de](mailto:tobias.gulder@ch.tum.de)

^7^Division of Genetic Engineering and Biotechnology, Department of Microbial Biotechnology, National

   Research Centre, El Buhouth St., Dokki, 12622 Giza, Egypt; Mohamed_M@klinik.uni-wuerzburg.de

* Correspondence: usama.ramadan@mu.edu.eg; Tel.: +20-86234-9075; Fax: +020-86237-6678

                                            tobias.gulder@ch.tum.de; Tel.: +49-89-289-13833


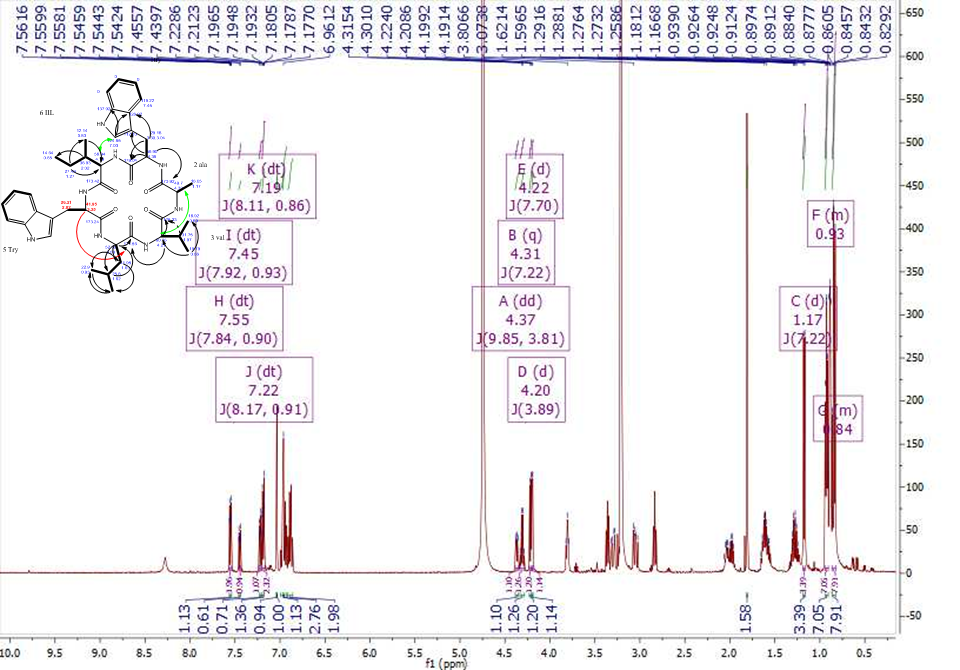


Figure S1:^1^H-NMR spectrum of compound 1 (nocardiotide A).


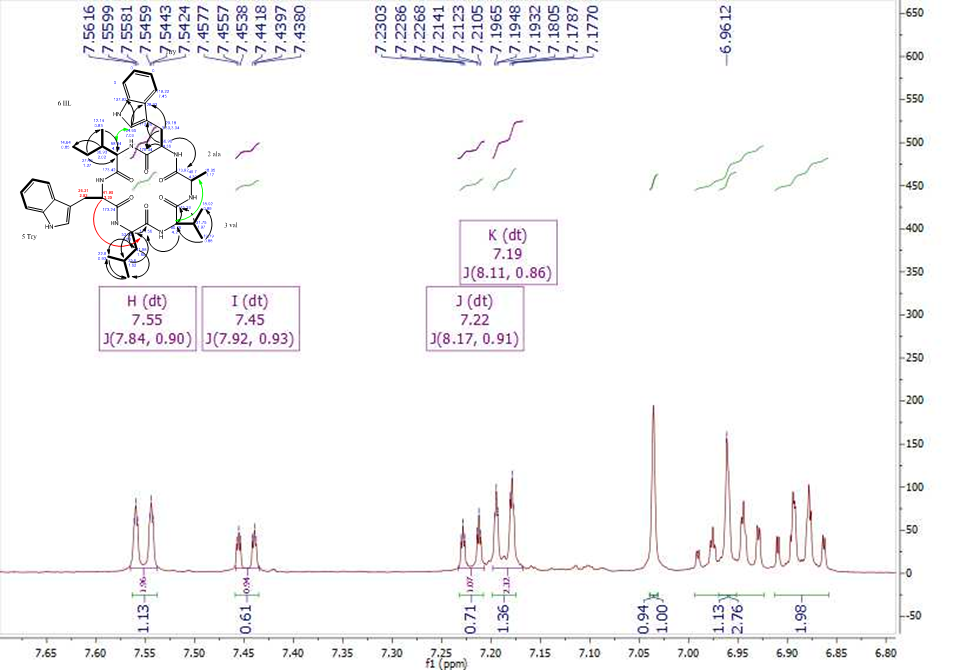


**Figure S2:Expanded downfield^1^H-NMR spectrum of compound 1 (nocardiotide A).**


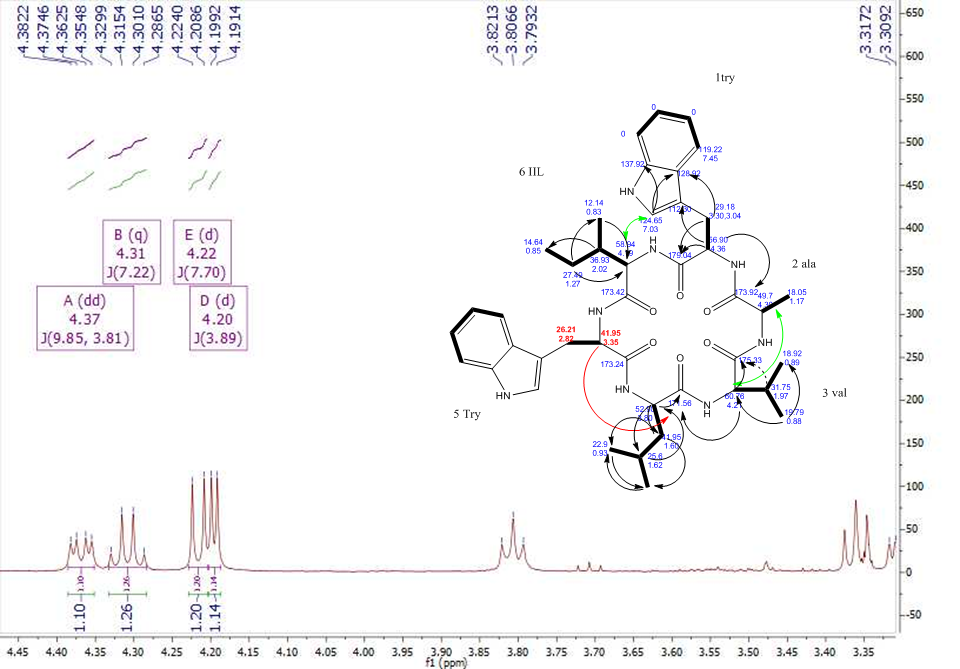


Figure S3:Expanded α-protons region^1^H-NMR spectrum of compound 1 (nocardiotide A).


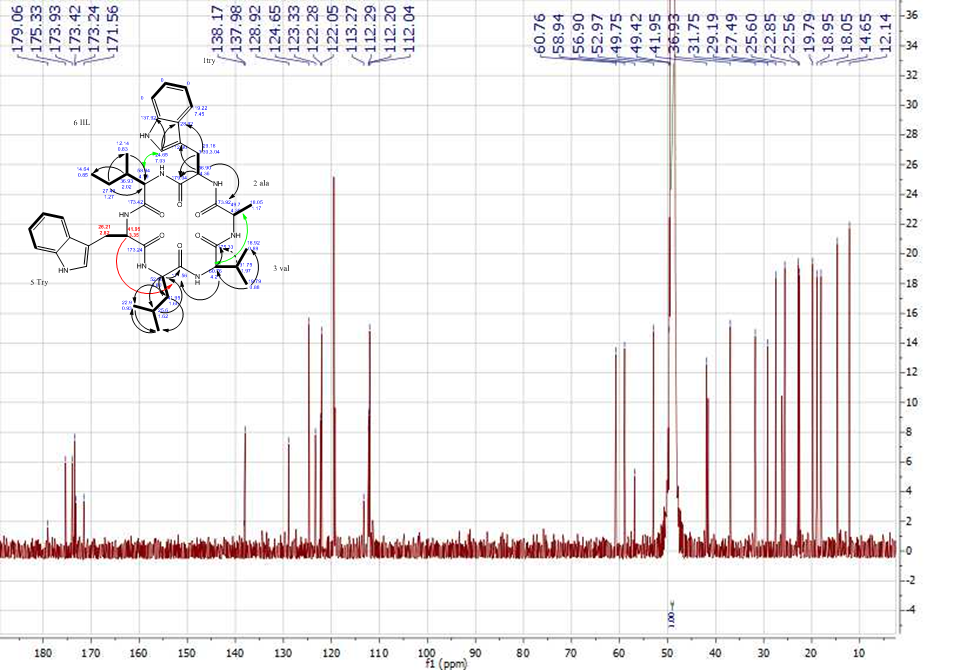


Figure S4:^13^C-NMR spectrum of compound 1 (nocardiotide A).


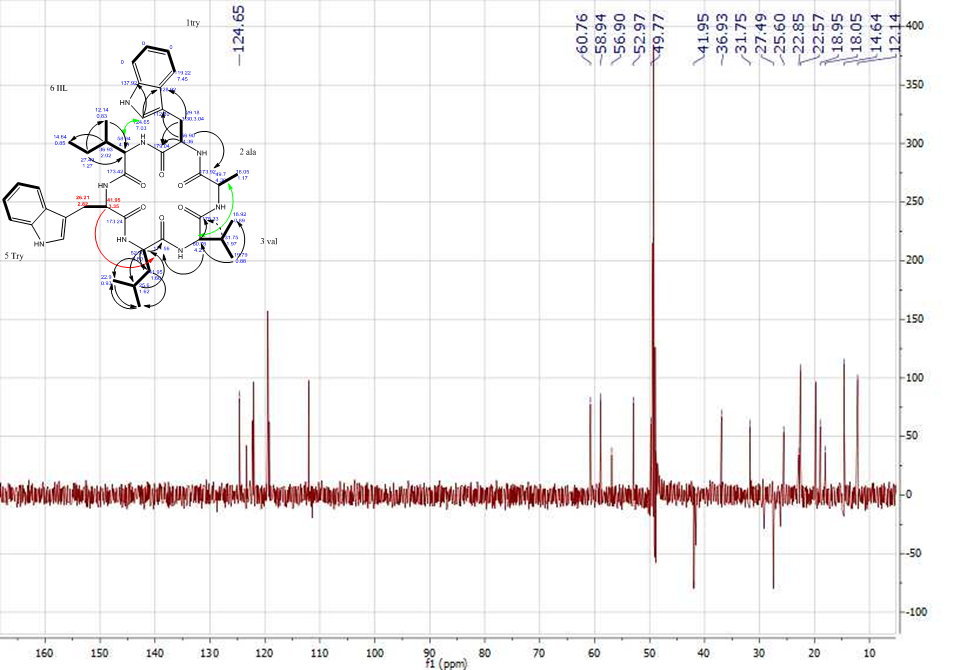


Figure S5:DEPT^13^C-NMR spectrum of compound 1 (nocardiotide A)


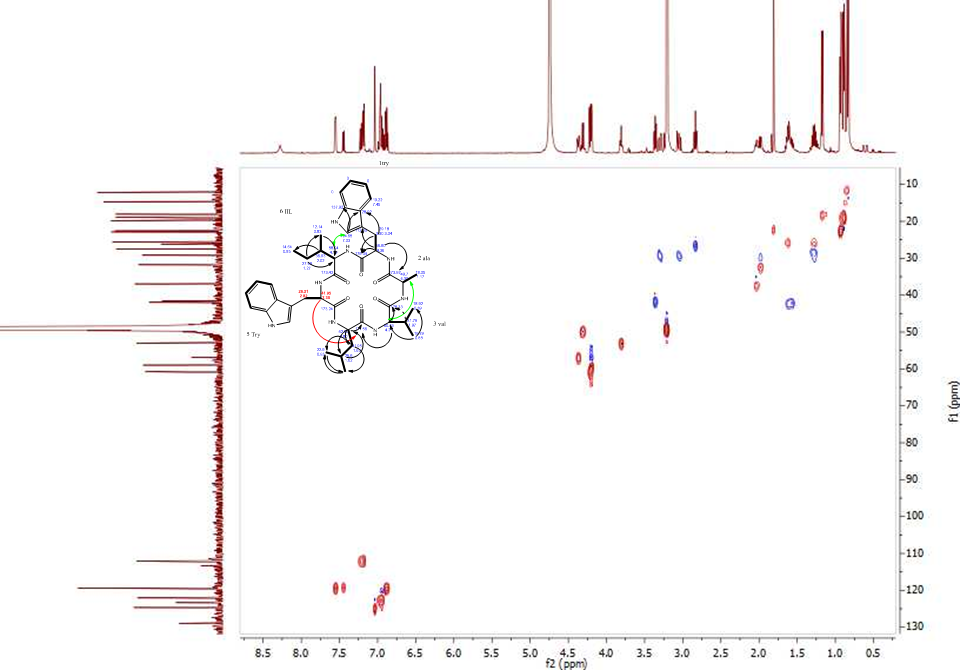


Figure S6:HSQC spectrum of compound 1(nocardiotide A).


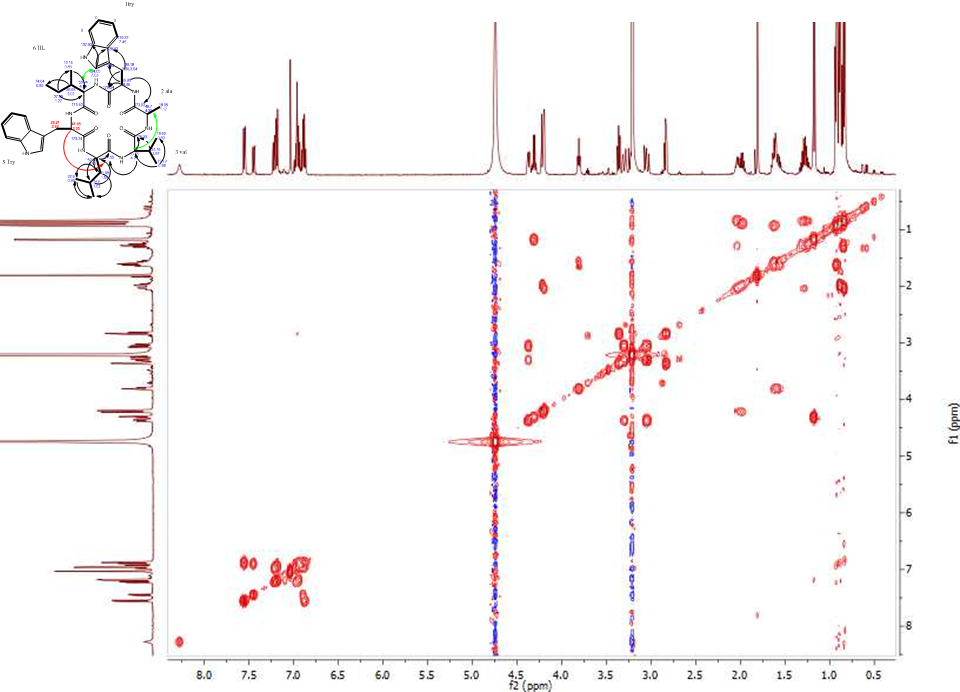


Figure S7:COSY spectrum of compound 1 (nocardiotide A).


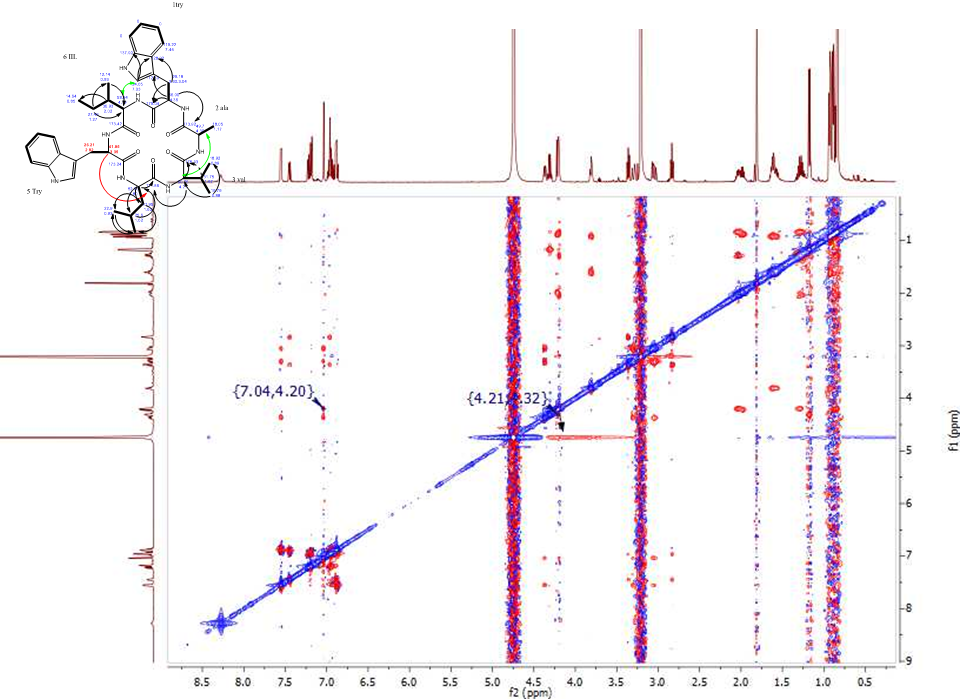


Figure S8:NOESY spectrum of compound 1 (nocardiotide A).


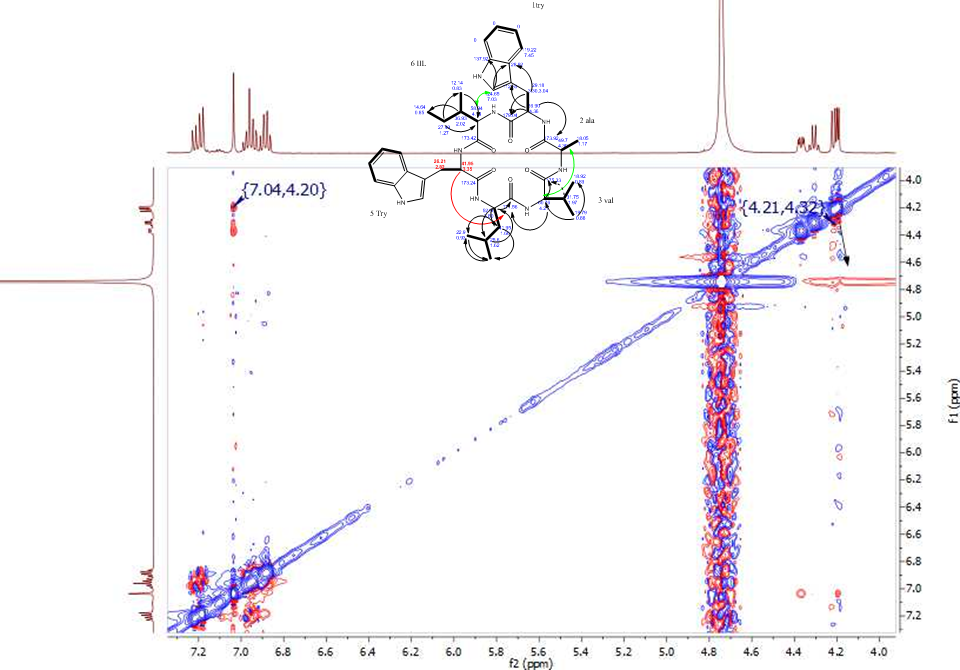


Figure S9:Expanded NOESY spectrum of compound 1 (nocardiotide A)


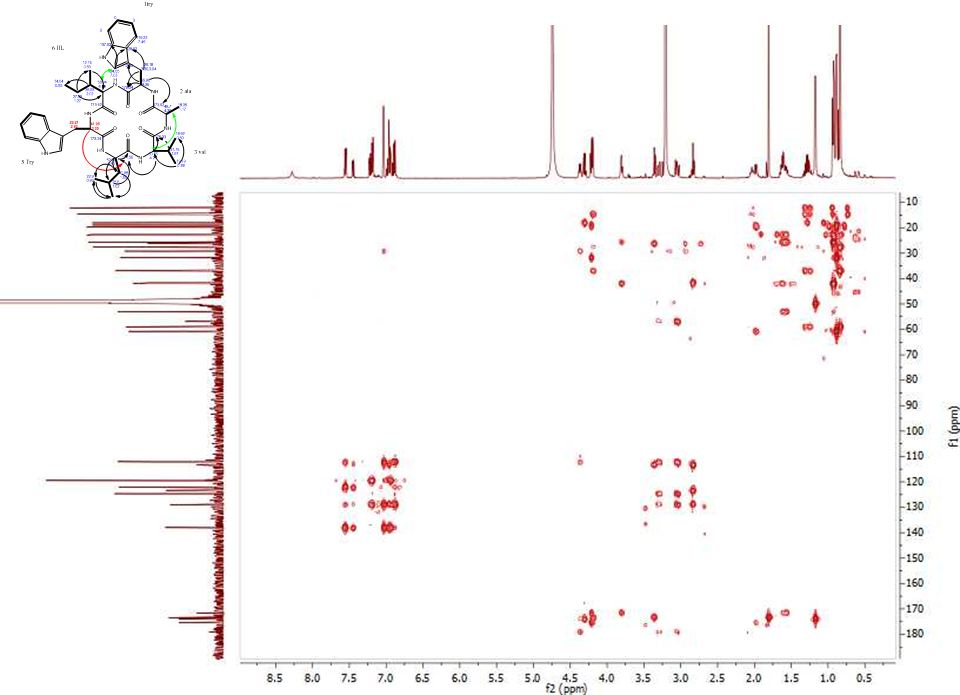


Figure S10:HMBC spectrum of compound 1 (nocardiotide A)..


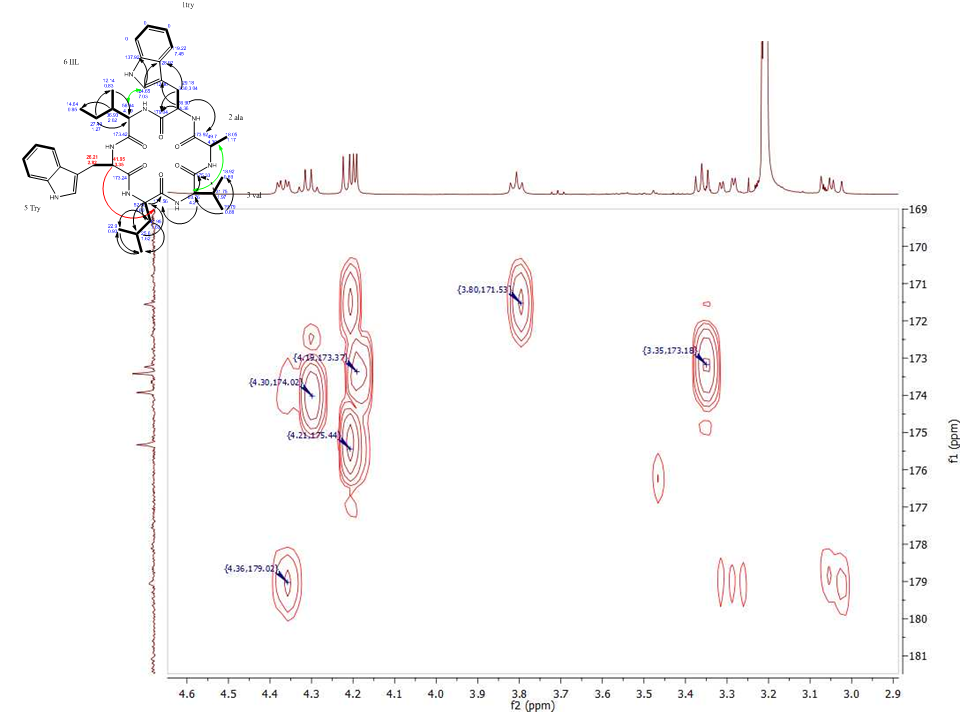


Figure S11:Expanded HMBC spectrum of compound 1 (nocardiotide A).


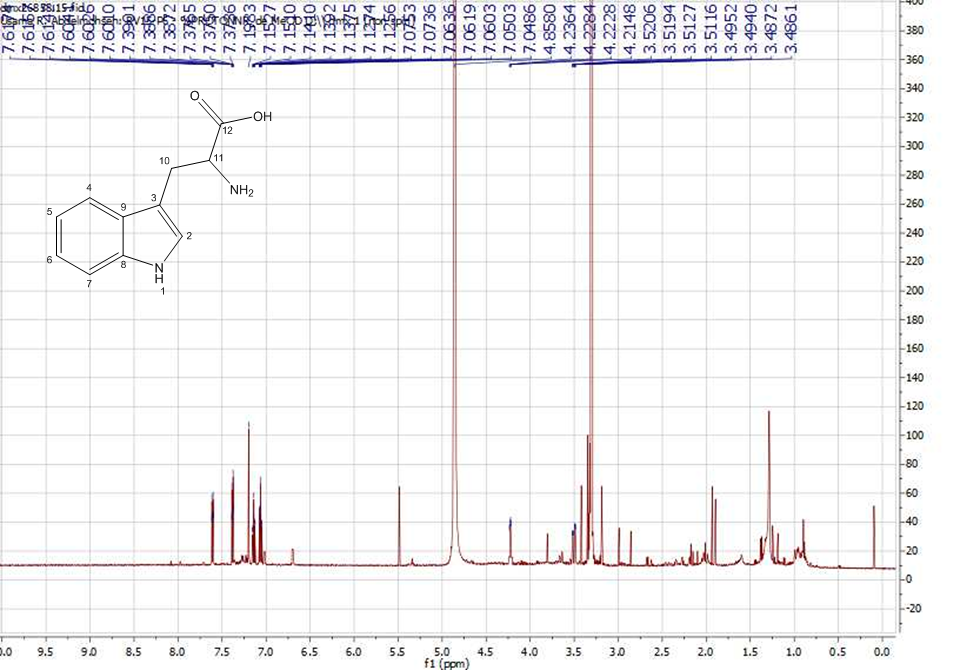
 Figure S12: ^1^H-NMR spectrum of compound 2 (tryptophan)


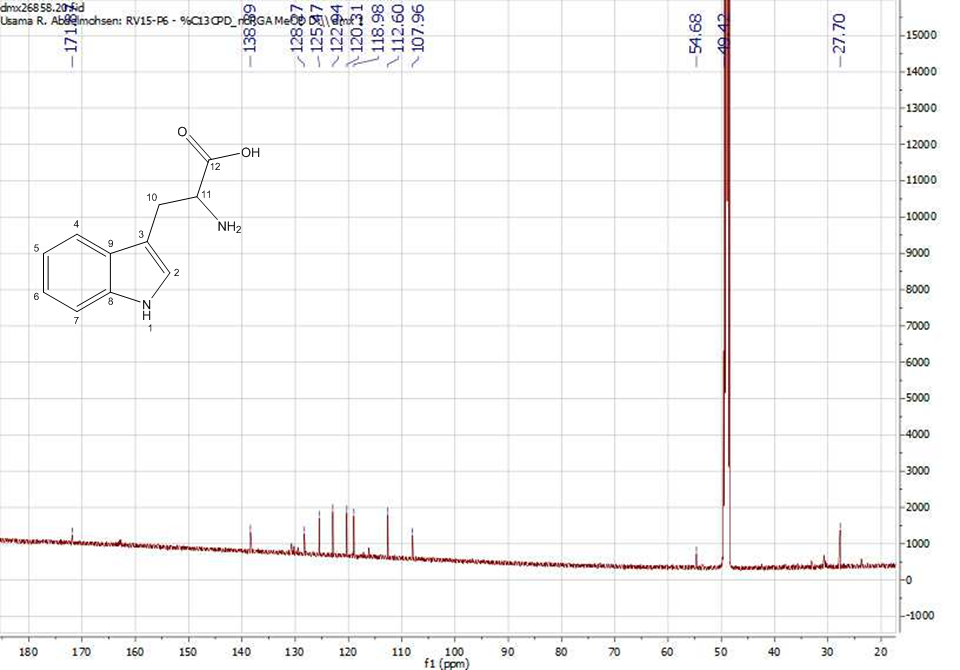


Figure S13: ^13^C-NMR spectrum of compound 2(tryptophan)

**
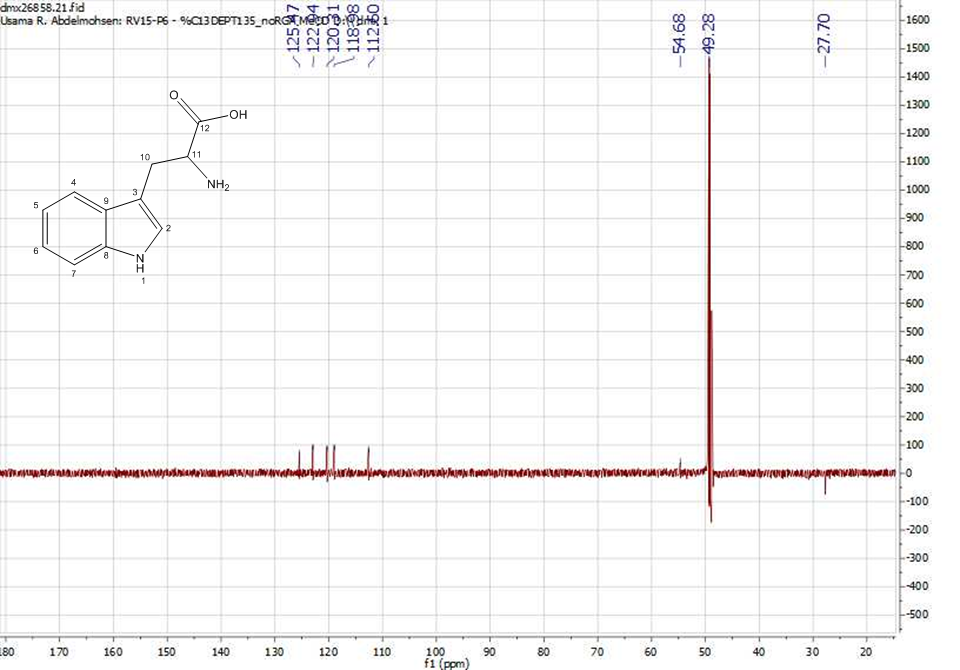
**

Figure S14:DEPT ^13^C-NMR spectrum of compound 2 (tryptophan)

**
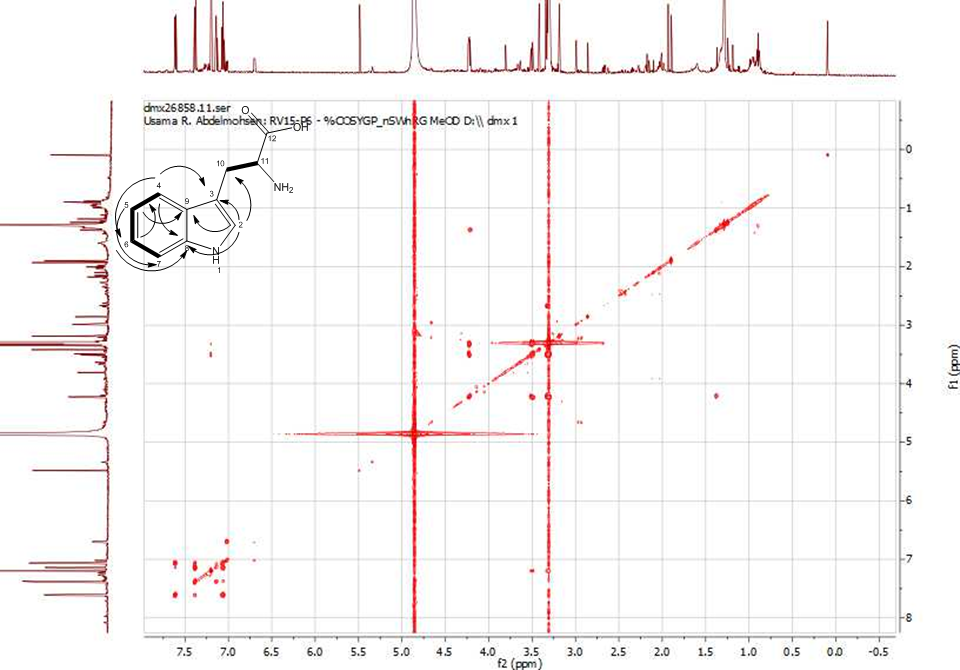
**

Figure S15: COSEY spectrum of compound 2 (tryptophan)

**
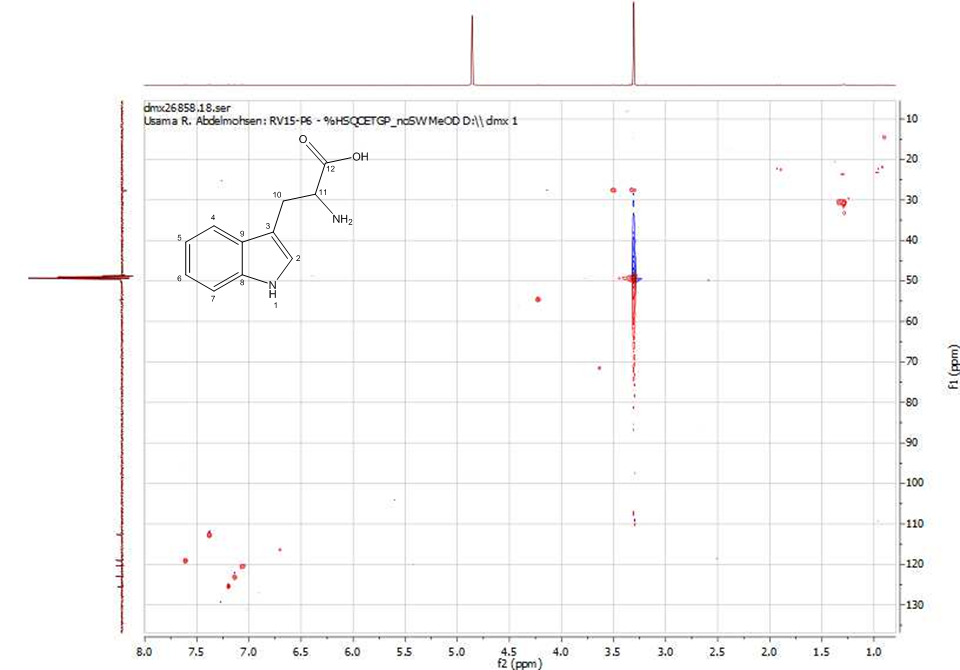
**

**Figure S16: HSQC spectrum of compound 2 (tryptophan)**


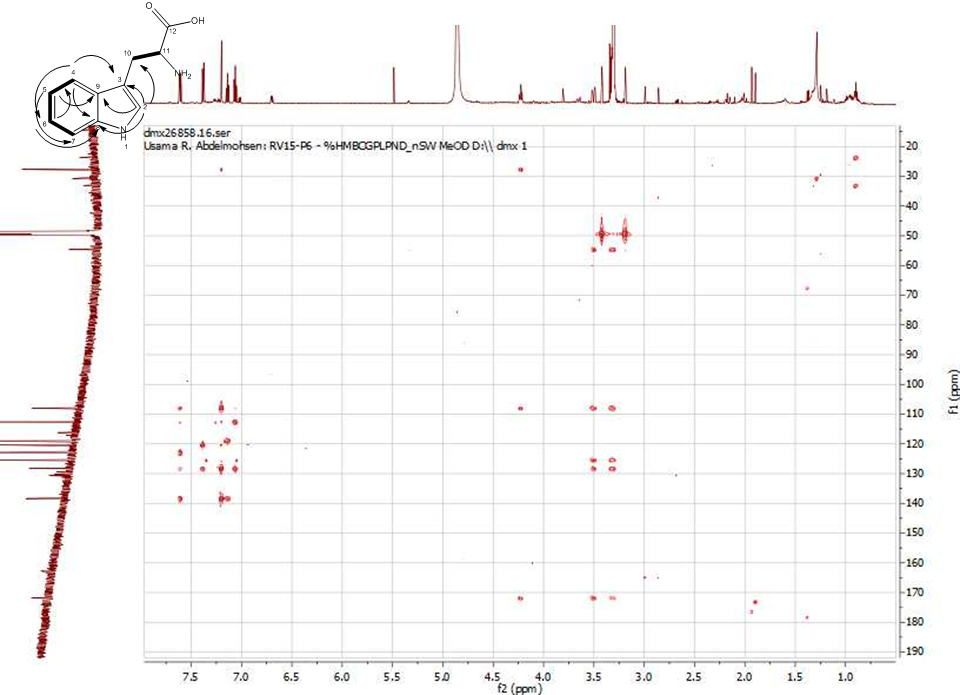


**Figure S17: HMBC spectrum of compound 2 (tryptophan)**

**
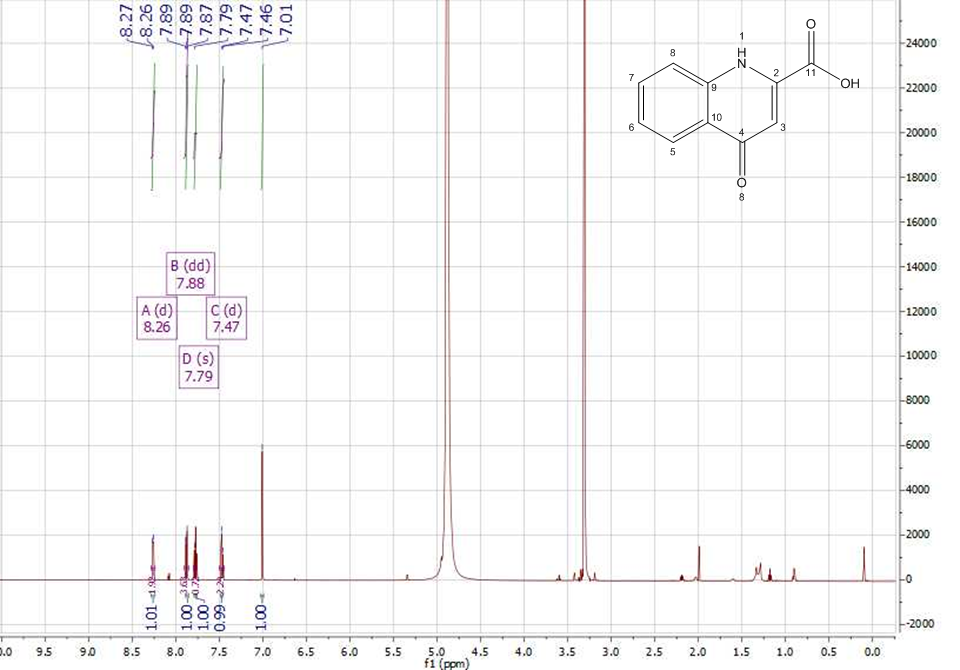
**

**Figure S18: ^1^H-NMR spectrum of compound 3 (Kynurenic Acid)**

**
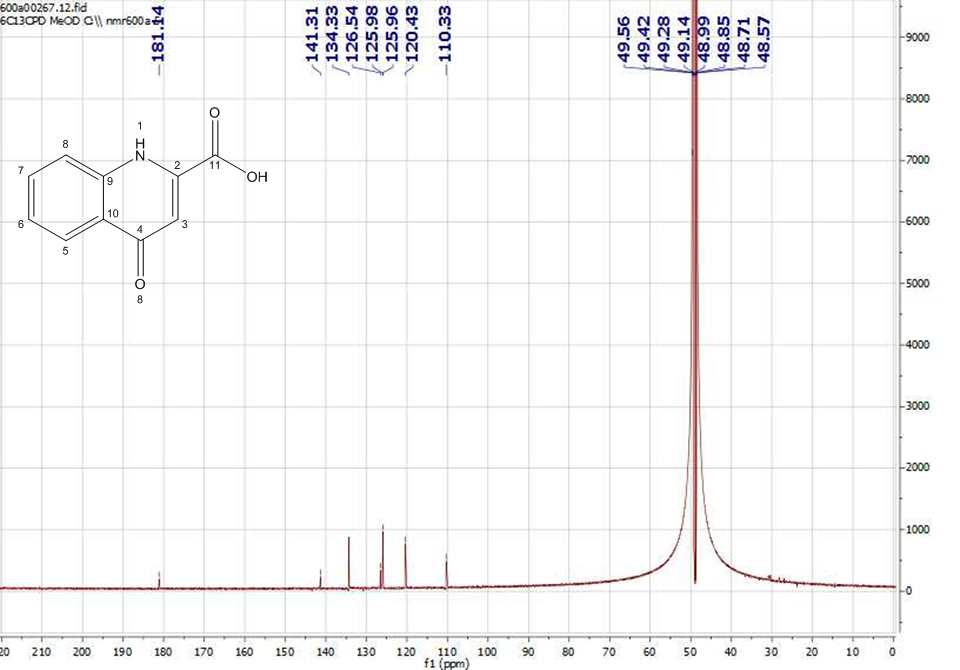
**

**Figure S19: ^13^C-NMR spectrum of of compound 3 (Kynurenic Acid)**

.

**
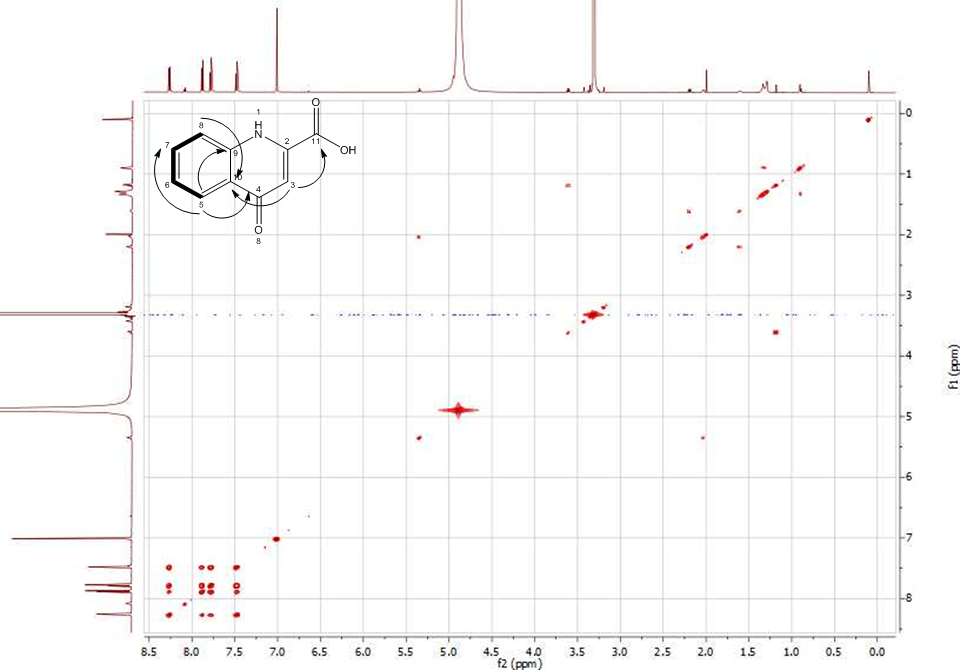
**

**Figure S20: : COSY spectrum of of compound 3 (Kynurenic Acid)**

.

**
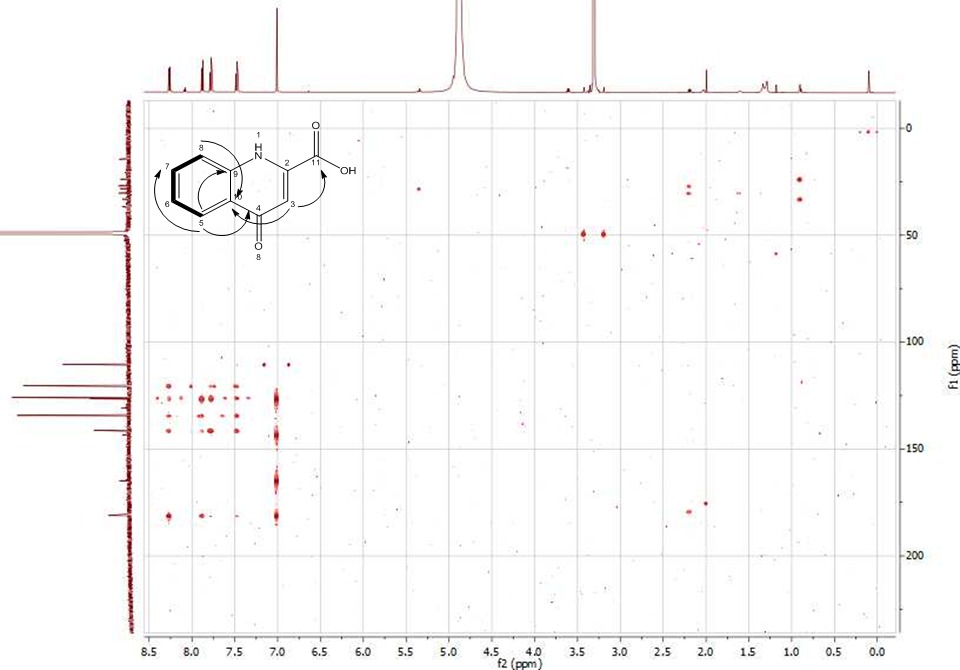
**

**Figure S21: : HMBC spectrum of compound 3 (Kynurenic Acid)**


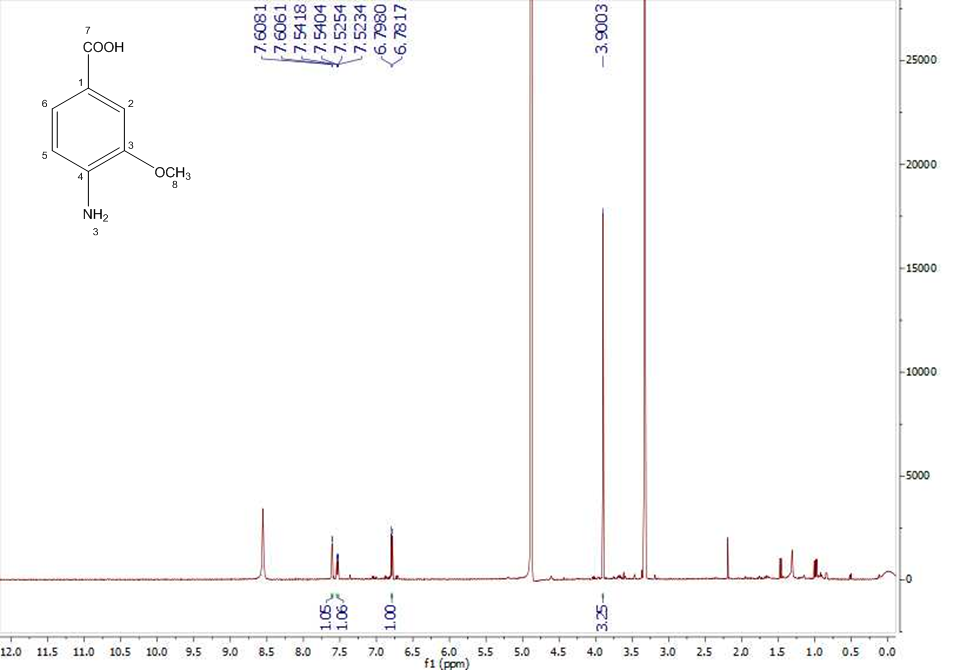


**Figure S22: : ^1^H-NMR spectrum of compound 4 (4-amino,3-methoxy benzoic acid)**


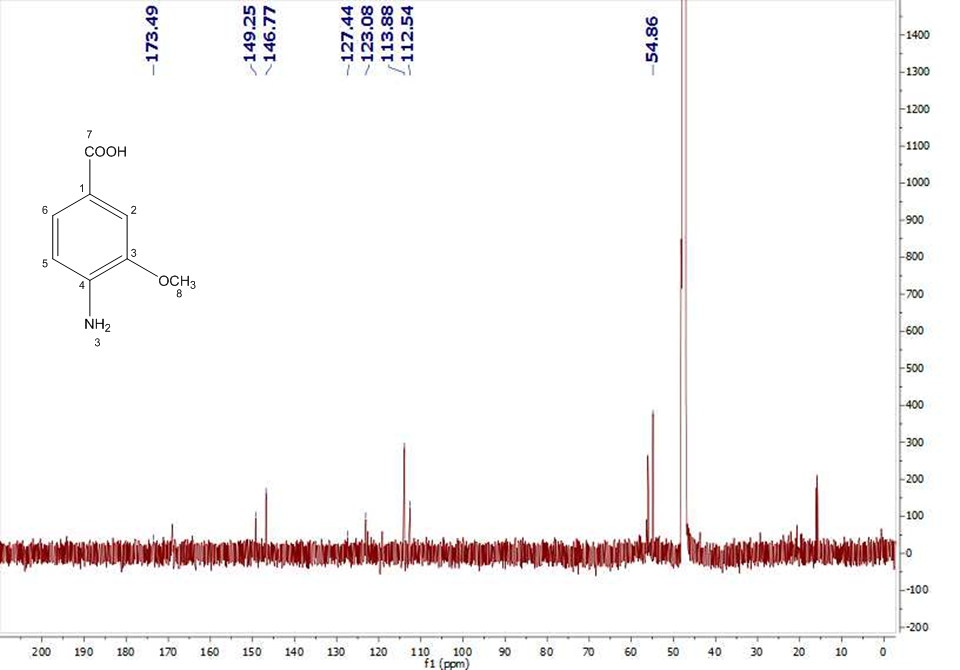


**Figure S23: ^13^C-NMR spectrum of compound 4 (4-amino,3-methoxy benzoic acid)**


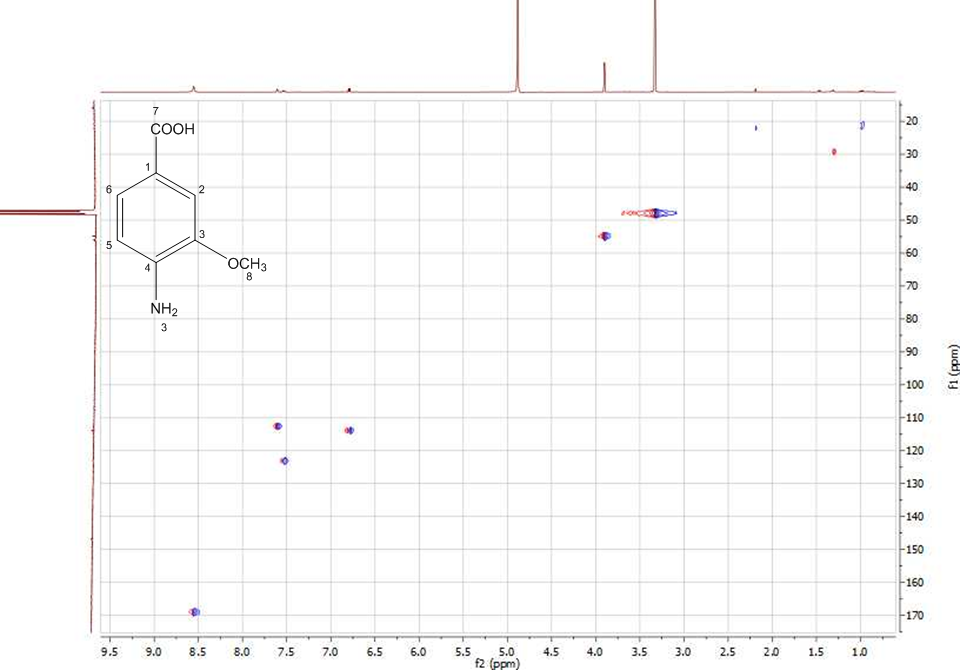


**Figure S24: HSQC spectrum of compound 4 (4-amino,3-methoxy benzoic acid)**


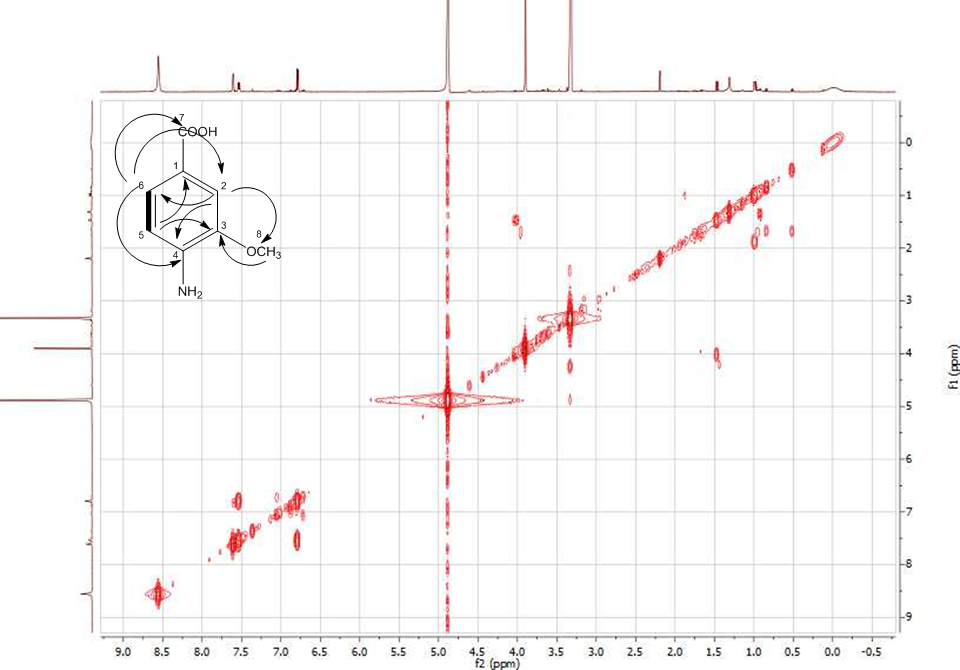


**Figure S25: COSY spectrum of compound 4 (4-amino,3-methoxy benzoic acid)**

.

.


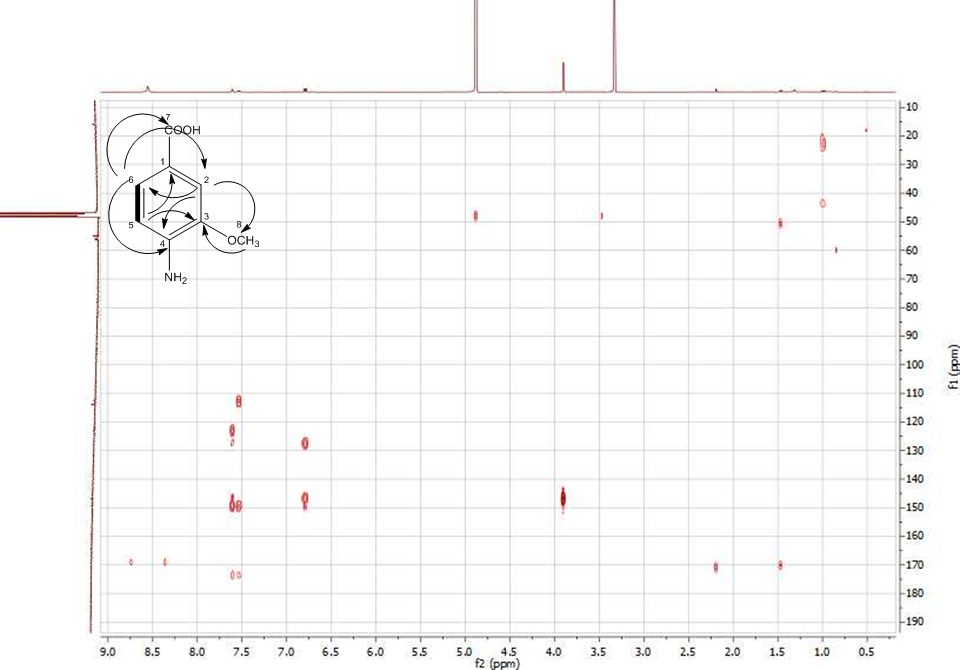


**Figure S26: HMBC spectrum of compound 4 (4-amino,3-methoxy benzoic acid)**
